# Up, Down, Near, Far: An Online Vestibular Contribution to Distance Judgement

**DOI:** 10.1371/journal.pone.0169990

**Published:** 2017-01-13

**Authors:** Ágoston Török, Elisa Raffaella Ferrè, Elena Kokkinara, Valéria Csépe, David Swapp, Patrick Haggard

**Affiliations:** 1 Brain Imaging Centre, Research Centre for Natural Sciences, Hungarian Academy of Sciences, Budapest, Hungary; 2 Institute of Cognitive Neuroscience, University College London, London, United Kingdom; 3 Department of Psychology, Royal Holloway University of London, Egham, United Kingdom; 4 Department of Personality, Assessment and Psychological Treatments, University of Barcelona, Barcelona, Spain; 5 Department of Computer Science, University College London, London, United Kingdom; Taipei Veterans General Hospital, TAIWAN

## Abstract

Whether a visual stimulus seems near or far away depends partly on its vertical elevation. Contrasting theories suggest either that perception of distance could vary with elevation, because of memory of previous upwards efforts in climbing to overcome gravity, or because of fear of falling associated with the downwards direction. The vestibular system provides a fundamental signal for the downward direction of gravity, but the relation between this signal and depth perception remains unexplored. Here we report an experiment on vestibular contributions to depth perception, using Virtual Reality. We asked participants to judge the absolute distance of an object presented on a plane at different elevations during brief artificial vestibular inputs. Relative to distance estimates collected with the object at the level of horizon, participants tended to overestimate distances when the object was presented above the level of horizon and the head was tilted upward and underestimate them when the object was presented below the level of horizon. Interestingly, adding artificial vestibular inputs strengthened these distance biases, showing that online multisensory signals, and not only stored information, contribute to such distance illusions. Our results support the gravity theory of depth perception, and show that vestibular signals make an on-line contribution to the perception of effort, and thus of distance.

## Introduction

Perceiving how far away an object is from one’s own body is essential for interacting with the environment. Distance can be inferred directly from visual information, using accommodation [1] and binocular cues such as vergence [2] and disparity [3]. However, distance perception is dramatically biased if the target objects are presented above or below the level of horizon. For example, a mountain refuge seems farther or closer depending on whether we look up at it from below or down at it from above [4]. Hence, purely visual information about distance may be affected by non-visual factors [5,6], such as fear of heights [4] or perceived effort of access [7].

Contrasting explanations have been proposed for non-visual *distance biases*. On the one hand, the *gravity theory* claims that distance perception is based on the estimated motor effort of navigating to the perceived object [7,8]. Accordingly upward distances are overestimated [9]. On the other hand, the *evolved navigation* theory posits an evolutionary advantage in overestimating the risk of falling [10,11]. On this view, contrary to gravity theory, downward distances are overestimated. Both theories assume that current head and gaze elevations are combined with internally-stored information in order to compute distance. Gravity theories require stored information about previous motor efforts [8], while evolved navigation theories require internal information about potential risks of falling [12]. Critically, removing the fear of falling by experimenting in low detail Virtual Reality [13] or reducing the expected effort of access by e.g. not wearing any heavy backpacks [9] reportedly diminishes these elevation distance biases.

In principle, the influence of upward/downward head inclination on distance perception could be based on *online* information, rather than stored information. In particular, under terrestrial conditions, the vestibular system constantly provides signals relating current head orientation to the direction of gravity. Combining a vestibular signal with an eye position signal specifies whether a visual object is located above or below the eye. Although vestibular signals do not directly code the spatial location of external objects, the interaction between vestibular and visual information is essential in providing the organism with space representation [14–16]. For instance, vestibular peripheral organs detect the motion of the head, producing experiences of self-motion in three-dimensional space. Cortical vestibular pathways integrate information from other sensory modalities to generate appropriate and accurate responses to self-motion, such as the stabilization of gaze, balance and postural motor commands [17], and the perception of the subjective visual vertical [18]. Microgravity experiments showed that visual perception of horizontal depth is influenced by altered vestibular signals [19]. For example, perceived distances were underestimated during either short-term exposure to microgravity using parabolic flight [20] or long term exposure on the International Space Station [21] leading to perceptual distortions of three dimensional space. However, the vestibular contribution to elevation biases in visual depth perception remains under-investigated.

Recent studies indicated that reaching an object can be affected by the posture of the body [6]. Tilting the body forward caused errors in the reaching movement, because participants underestimated the distance between their own body and the virtual object. These results seem to support the gravity based model. Similarly, Harris and Mander [5] reported that tilting the body backward caused overestimation of the perceived length of a rod, and hence, the wall seemed presumably closer. However, these studies do not specify under which circumstances one expects underestimation vs. overestimation, and why.

Here we asked participants to judge the distance of an object presented at different distances on an inclined plane, leading to different head and gaze elevations. We developed a novel Virtual Reality environment in which neither risks of falling [22], nor navigational effort were actually present [23]. This minimised confounds such as familiarity, and memory for previous efforts, that could affect previous field-based experiments. The participants’ head inclination was systematically varied by asking them to tilt both the head and gaze upwards or downwards to fixate a target object. We could thus directly compare predictions of gravity and evolved navigation theories. Further, we applied event-related galvanic vestibular stimulation (GVS) during each judgement, to investigate whether online vestibular signals indeed affected the distance perception biases. Importantly, GVS is a non-invasive method that directly stimulates the vestibular receptors [24], producing complex oculomotor, perceptual and postural responses. In the traditional bilateral bipolar GVS configuration, an anode and cathode are placed on the left and right mastoid, or vice versa. Perilymphatic cathodal currents are thought to depolarize the trigger site and lead to excitation, whereas anodal currents hyperpolarize it resulting in inhibition [25]. This is considered to enhance the vestibular activity by mimicking a natural movement of the head, which elicits a virtual sensation of roll tilt.

## Methods

### Ethics Statement

The experimental protocol was approved by the local ethics committee (University College London) and the study was conducted in line with the Declaration of Helsinki. Participants gave written informed consent to participate in the experiment before inclusion in the experiment.

### Participants

Sixteen healthy participants volunteered for the study. Data from two participants was discarded because they proved unable to follow the instruction of the experiment (see below). Thus, fourteen participants (5 females, mean age ± standard deviation: 26.64 ± 6.64 years) completed the experiment. All participants were right-handed according to their Edinburgh handedness inventory scores. The sample size was decided *a priori* based on similar experiments [5,6].

### Galvanic Vestibular Stimulation

**Fig 1:**
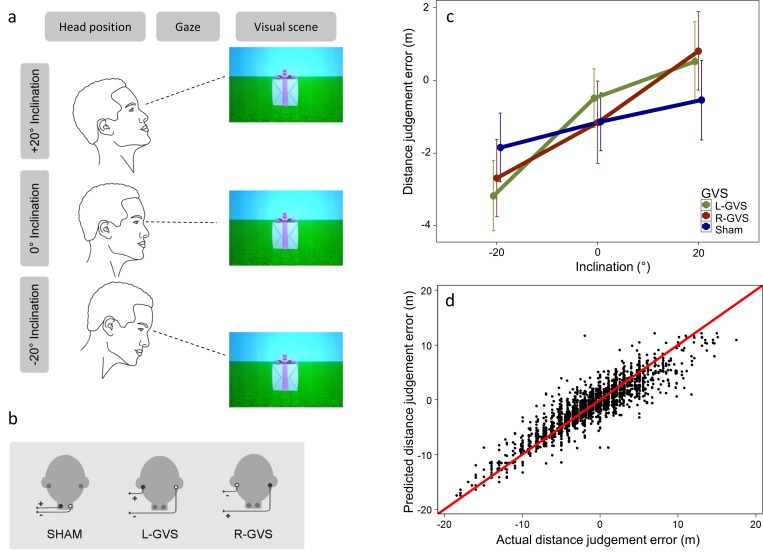
Experimental set up and results. (a) Participants were seated in the centre of the cave. During the experiment, participants made absolute judgements of the distance between their own body and an object (a gift box) appearing in front of them. The positions of the gift box were distributed logarithmically between 5m and 25m. The same distances were presented with the three head inclinations -20°, 0°, and +20°. The gaze was aligned with head inclinations. (b) Left anodal and right cathodal configuration is named ‘L-GVS’. The inverse polarity, namely right anodal and left cathodal configuration, is named ‘R-GVS’. A sham stimulation was also applied placing the electrodes to the left and right side of the neck about 5cm below the GVS electrodes. GVS and sham stimulation were applied delivering a boxcar pulse of 1ma for 3s. (c) Distance errors have been calculated by subtracting the actual distance from the judged distance. Estimation bias in 0° head inclination condition was used as baseline, and all values were corrected by this baseline. Thus, negative values on the ordinate indicate underestimation compared to the horizontal, zero-inclination baseline condition, whereas positive values indicate overestimations. Distance perception varied significantly according head inclination. Specifically, downward distances were underestimated, while upward distances were overestimated, relative to baseline. This pattern of distance illusions is in line with the predictions of the gravity theories. Note that GVS enhances this pattern. (d) Predictions based on linear mixed-effects model. The model containing both fixed and random terms fits well to the actual data.

Bipolar galvanic vestibular stimulation (GVS) was applied using a commercial stimulator (Good Vibrations Engineering Ltd., Nobleton, Ontario, Canada) delivering a boxcar pulse of 1mA for 3s. This low intensity was used to minimise non-specific cueing effects, such as arousal from cutaneous sensations or vertigo. Importantly, several studies confirm that this level of GVS activates the vestibular organs. For instance, both postural [24] and behavioural changes have been reported with such low intensities GVS [26–30]. GVS is known to increase the firing rate in vestibular afferents on the cathodal side and to decrease the firing rate on the anodal side [25], enhancing the ongoing natural vestibular responses and evoking virtual sensations of roll tilt. These effects do not outlast the stimulation [31]. Carbon rubber electrodes (area 10 cm^2^) coated with electrode gel were placed binaurally over the mastoid processes and fixed in place with adhesive tape. The area of application was first cleaned, and electrode gel was applied to reduce the impedance. Left anodal and right cathodal configuration is named ‘L-GVS’ (Fig 1B). The inverse polarity, namely right anodal and left cathodal configuration, is named ‘R-GVS’. We also applied a sham stimulation using electrodes placed on the left and right side of the neck, about 5cm below the GVS electrodes [32,33] with left anodal and right cathodal configuration. This sham stimulation evoked similar tingling skin sensations to GVS but not modulation of vestibular afferents. It thus provided a control for non-specific alerting effects and for the knowledge that an unusual stimulation is occurring.

### Virtual Reality Environment

The experiment was carried out in the Immersive Virtual Reality Laboratory at University College London, using a CAVE facility [34]. This system consists of four stereo-projected surfaces: three back-projected vertical walls, each 3m wide x 2.2m high, and the floor (3m x 3m) form a continuous projection surface. The Virtual Reality environment was created using Unity3D game engine (www.unity3d.com), rendered using a K5000 graphics card to drive 4 Christie Mirage DLP projectors, each of which projected to one of the 4 screens at 96Hz. The participant wore shutter glasses synchronized with the projectors creating active stereo-projection in each eye at 48Hz. The glasses provided a field of view per eye of approx. 90–100° horizontally and 60–70° vertically (the precise field of view depends to some extent on how closely the glasses fitted to the participant’s eyes). The position of the glasses was tracked by an InterSense™ IS-900 system with high accuracy. The system was calibrated to the participant’s own eye height at the beginning of every experiment, and this data was used to accurately compute object distances for the upward, downward, and level inclinations. This calibration was performed to account for small changes in eye-to-ground distance among participants. A geometrical model accounting for the pitch of the ground plane and the eye-height of the participant ensured that the distance from the participant's eye to the near-top edge of the target object was identical for both upward and downward pitched objects (as well as among participants with different eye-heights). The virtual scene was a green grass-like plane with blue skies and no visible landmarks. The experimental object was a 2m X 2m gift box with purple ribbon (see Fig 1A). The object rested on the ground and the same proportion of object and environment was visible at all inclinations. The unusual size of the target object was chosen to be appropriate for the range of distances presented in the experiment and the consequent visual angle subtended by the target object at these distances. As the distance was varied between 5m and 25m, and the object had 2m sides, the visual angle subtended varied between 23° and 4.6°.

### Experimental Procedure

Verbal and written instructions about the task were given to participants prior to the experiment. Participants were seated in the centre of the CAVE, 1.5m from the front screen. A visual scene was presented on vertical screens and on the floor in order to create a seamless, wide field-of-view immersive display. Participants made absolute judgements about the distance between their own body and an object (a gift box) that appeared in front of them ([35], see Fig 1A). At the beginning of the task, the target object was displayed for few minutes to allow participants to familiarise with its size. Then object positions slightly under (1.5m) and slightly over (30m) the experimental range were presented, and the experimenter informed the participant about the actual distance in metres.

Participants were encouraged to use these as anchor points to calibrate distance judgements in experimental trials. The positions of the present box were distributed logarithmically between 5m and 25m; thus, the possible distances were 5, 6.9, 9.52, 13.3, 18.2, 25m. These distances were chosen to produce a wide range of perceived distances. Our predictions did not focus on the effects of object distance itself but rather on the effects of two other experimental factors: head inclination and vestibular stimulation. Participants were instructed to look horizontally relative to the head and visual ground plane, but the angle of the head was manipulated across experimental conditions. The object appeared on a smooth plane that was inclining (+20°), flat (0°), or declining (-20°), and participants were asked to tilt their head accordingly (backwards, natural and downwards). The experiment was divided into blocks; head inclination (+20°, 0°, -20°) and vestibular stimulation (L-GVS, R-GVS, Sham) changed only between blocks. Each block consisted of 18 trials; there were three repetitions of the same distance in each block. Distances were presented in random order. Block order followed a Latin square design. Each trial started with the presentation of the grass-like plane in the actual inclination and the blue sky. Participants adjusted their head pitch angle to fixate the object and, therefore, the horizon, while a 6 degrees head tracking system monitored their posture. This procedure ensured that participants saw the same proportion of grass and sky at all head inclinations. The head tracking system measured the inclination of the head and a sound signalled when the participant’s head reached the correct vertical angle. Thus, the head position in space was decoded by our custom-built software for presenting stimuli, which started each trial if and only if participants’ head was in the correct inclination. Participants were told to keep their head at the same position for the duration of the block. Then GVS/Sham started and lasted for 3s. 1s after GVS onset, the gift box became visible for 1s and then disappeared. This delay was used to ensure that vestibular cortical projections would be activated when visual stimulus was present: Fitzpatrick and Day [24] reported that 1s of 1mA GVS produced clear postural adjustments in standing participants, implying successful activation of the vestibular system. The image was then blurred, and the GVS/Sham pulse ended. Participants made absolute verbal judgements (in metres) of the distance of the object after the screen was blurred. The response was recorded, and the next trial started. This method of reporting distance percepts has high face validity, and allows many estimates to be acquired rapidly. It was thus preferred to the method of limits and method of constant stimuli favoured in classical psychophysical studies. Very importantly, any imprecision or bias resulting from this method of measurement should affect all GVS conditions equally. We did not aim to quantify the limits of visual distance perception, but only to compare estimates of perceived visual distance between GVS conditions. We wanted to sample a range of environmental distances to minimise the number of GVS stimulations (GVS can cause mildly unpleasant sensations) and to minimise duration of the CAVE immersion. Absolute judgements might be criticised because different participants may use different subjective standards. However, our experimental design was based only on within-participant comparisons; therefore, differences between individuals in reported values do not affect our inferences.

## Results

Trials containing either recording errors or multiple responses were eliminated before the analysis. Less than one percent of all participants’ data was removed according to this criterion. We calculated the *distance judgement errors* by subtracting the simulated distance from the judged distance (see S1 File). We inspected the distribution of errors expressed both in metres, and as a percentage of the actual distance. The former distribution was normal, whereas the latter was left-skewed. We therefore preferred to express errors in physical units (m). The distance bias in the 0° head inclination condition, corresponding to horizontal gaze, was been considered as baseline, which allowed us to define underestimation (negative values) and overestimation (positive values) relative to it.

First, distance judgement errors for each participant were averaged for each combination of head inclination and vestibular stimulation conditions and analysed using factorial repeated measures ANOVA and planned contrasts. Our theoretical predictions focused on the interaction between head inclination and vestibular stimulation factors. These analyses therefore pooled across the different distances judged. Distance perception varied significantly across head inclinations (*F*(2,26) = 23.694; *p <* .001; *η*_*p*_^*2*^ = 0.65) (Fig 1C). Overall estimations showed a slight underestimation trend, but only estimations in the -20° head inclination condition were significantly underestimated compared to actual distances (*t*(13) = -2.75, p = .02, *Cohen’s d* = -1.52, all other *p*> .3). Downward distances were underestimated by 1.65m (*SD* = 3.50), while upward distances were overestimated by 1.19m (*SD* = 3.90), compared to ground level. This pattern of results fits the predictions of gravity theories but opposes the predictions of evolved navigation theories. A planned linear trend contrast confined to the sham condition also showed a trend in the direction predicted by gravity theories (down vs. up head inclination *t*(1,13) = 1.670; *p =* .059, *Cohen’s d* = 0.45, one-tailed, numerical effect present in 10/14 participants). The corresponding planned contrast for evolved navigation was not supported (flat vs down head inclination: *t*(1,13) = -1.274, n.s.). The main effect of vestibular stimulation was not significant (*F*(2,26) = 0.196; *p* = .823). However, and more importantly, we found an interaction between vestibular stimulation condition and head inclination (*F*(4,52) = 3.318; *p* = .017; *η*_*p*_^*2*^ = 0.20). This interaction occurred because the linear trend predicted by gravity theories was amplified by both polarities of GVS (down vs. up head inclination L-GVS *t*(1,13) = 4.891, *p* < .001, *Cohen’s d* = 1.31; R-GVS *t*(1,13) = 6.585, *p* < .001, *Cohen’s d* = 1.76, numerical effect averaged across GVS polarities present in 14/14 participants). This pattern of results is consistent with an inclination effect generated online by a vestibular signal that is boosted by artificial vestibular stimulation.

Second, we fitted a mixed effects model to investigate intersubject variability, following Barr et al [36]. In this mixed effects approach, we entered head inclination (-20°, 0°, 20°) and object position as scalar variables [37], while vestibular stimulation (L-GVS, R-GVS, Sham) was handled as factorial. Mixed effects modelling was performed in R [38] using lme4 [37]; ggplot2 was adopted for visualisation [39].

**Fig 2:**
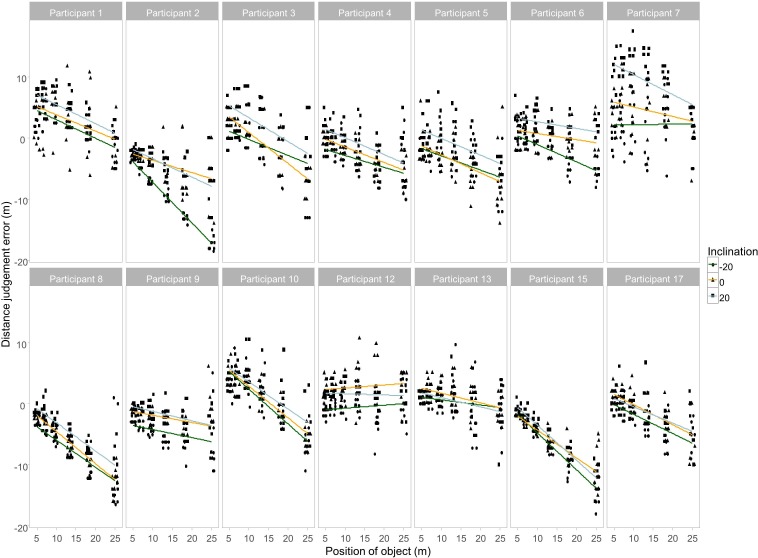
Relation between object position and judgement error in individual participants. See text for explanation. Note that the size and even direction of the relation differs between participants.

We inspected how distance judgement errors varied as a function of object position for each participant (Fig 2). We observed a strong correlation between object distance and judgement error within most participants, together with strong differences between participants in the strength and even the sign of these correlations. This pattern of variation justifies the explicit modelling of both fixed and random effects provided by the mixed model approach [36]. We included both fixed (i.e. population general) and random (i.e. subject specific) effects of head inclination, object position and vestibular stimulation [37,40]. We aimed to keep maximal random effect structure in the model [36], therefore random intercepts were estimated for individual subjects, individual subject and vestibular stimulation combinations, individual subject and inclination combinations, and individual subject, vestibular stimulation and inclination combinations. Random slope is only estimated for the object position, but separate random slopes (correlated with their respective intercepts) were estimated for different random intercept terms. For this model the restricted maximum likelihood (RMEL) estimation reached convergence. This model appeared to fit our data well according to the Akaike Information Criteria (AIC) of 3243.747 (df = 31, baseline model containing no fixed effects, and only the subjects as random effects resulted in AIC 5211.152). The fixed effect significances were tested using *F* tests, where *p* values were based on the Kenward-Roger approximation of the degrees of freedom [41]. We found a significant main effect of head inclination (*F*(2, 26.17) = 23.70, *p* < .001, *η*_*p*_^*2*^ = 0.64) and a main effect of object position (*F*(1, 19.87) = 41.75, *p* < .001, *η*_*p*_^*2*^ = 0.68). Critically, the interaction between head inclination and vestibular stimulation was significant (*F*(4, 52.92) = 3.68, *p* = .010, *η*_*p*_^*2*^ = 0.22). The pattern of interaction was consistent with an involvement of vestibular gravitational signals in distance estimates, with the effects of actual distance on reported distance being greater with GVS than in Sham. Least square means post hoc contrasts revealed significant differences between -20° and 20° head inclination conditions in the Sham (*p* = .035). Finally, the three way interaction between head inclination, vestibular stimulation, and object position was also significant (*F*(4, 67.56) = 39.10, *p* < .001, *η*_*p*_^*2*^ = 0.70), primarily due to changes in slopes estimated for some head inclination and vestibular stimulation combinations. The overall model explained more than 80% of total variance in the data (*Conditional R*^*2*^ = 0.82) with a compelling contribution of the fixed effects (*Marginal R*^*2*^ = 0.23) (Fig 1D). The difference between conditional and marginal R^2^ indicates how much variance is present on the subject level and captured by the random effects in our model. This suggests that our mixed effects model fitted well with the observed data, and validated hierarchical modelling of the experimental variable.

To summarise, the ANOVA and the mixed effects model showed converging results. Downward distances were underestimated, whereas upward distances were overestimated compared to estimations made on flat surface. Furthermore, GVS enhanced these biases.

## Discussion

Participants overestimated visual distances when their head was tilted upward and underestimated them when their head was tilted downward, compared to judgements made in a zero-degree, horizontal baseline condition. The observed effect is in the direction predicted by gravity theories, but opposite to the predictions of evolved navigation theories. More strikingly, the distance biases increased strongly with event-related GVS. Our results suggest that the gravitational modulation of visual distance perception depends on on-line vestibular signals. This elevation distance bias is, therefore, not merely a product of learned contextual associations but rather reflects a specific multisensory integration mechanism.

Gravitational signals are coded by vestibular receptors in the inner ear, whose signal depends on the position of the head relative to gravitational vertical [42,43]. The precise mode of action of GVS remains debated, but recent evidence confirms activation of both otolithic fibres and semicircular canals [44]. In the bilateral bipolar GVS configuration, perilymphatic cathodal currents are thought to depolarize the trigger site and lead to excitation, whereas anodal currents hyperpolarize it resulting in inhibition [25]. Neuroimaging studies using GVS have revealed widespread vestibular projections reaching many areas of the cerebral cortex, such as the retroinsular cortex, the superior temporal gyrus, the temporo-parietal cortex, the basal ganglia and the anterior cingulate [45,46]. Critically, recent studies suggested that otolithic gravitational inputs in the vestibular system have a direct influence on cognitive tasks involving three-dimensional perception [5,6,14,15]. In particular, perception of depth was altered in microgravity and in peripheral vestibular disorders [15,47]. However, these results were attributed to changes in visual linear perspective and visual size perception, which should be specific to visual horizontal stimuli. Our data suggest an alternative mechanism for these effects. We found that artificial activation of vestibular projections in the brain by GVS modulated distance illusions. The pattern of modulation suggests that GVS amplified the neural signals generated by head and gaze elevation changes required to fixate targets above or below the ground plane [48,49]. Our results therefore suggest that vestibular inputs contribute to an on-line representation of head movement and position which is used in estimation of visual distance.

Gaze position and the perceived orientation of the ground plane are already known to contribute to perceived depth [49,50]. Critically, these factors both depend on head position, suggesting a fundamental interaction between visual, vestibular and proprioceptive signals in computing distance estimates. Combining traditional visual cues to depth, such as accommodation and vergence, with vestibular signals about current head position relative to gravity provides sufficient information to compute a possible motion path to a visual object [17], as suggested by navigation theories. In our study, participants overestimated distances when their head was tilted upward and underestimated them when their head was tilted downward compared to estimates in the horizontal plane. This distance perceptual bias was enhanced with event-related GVS. Interestingly, GVS did not interfere with distance perception when head inclination was zero. Presumably, when the head is level and not inclined, the brain computes distances to visual targets with respect to an assumed level ground. This represents an intermediate, neutral situation where there is neither cost nor benefit of gravity (cf [7,8,51]). In this special case, the online vestibular-gravitational signal generated by GVS does not need to be integrated.

Importantly, although the current results supported the predictions of the gravity theory, we do not suggest a globally linear relation between head inclination and distance error. In fact, not all possible angles are equally experienced in real environments [9,52]. Our experience of inclines typically involves either slight elevations (e.g. road gradients are usually under 20 degrees) or risky, non-navigable surfaces (e.g. the sheer drop from a balcony). One might expect the biases of evolved navigation theory to be most apparent for dramatic elevation angles associated with dangerous environments.

Verbal reporting of absolute distance judgements was used in the present study. This does not figure among the classical psychophysical methods. However, it has been used earlier [53,54]. The results of those studies were broadly similar to others that used other measures to assess distance estimation [55–57]. The verbal method has the advantage of being extremely rapid. This is important in environments such as VR, where long exposure is uncomfortable and impractical. Although absolute response accuracy may be low, this need not obscure the difference between our experimental conditions [58–61]. Thus, it seems very unlikely that the particular features of this psychophysical judgement can explain our results, unless additional and unwarranted ad hoc assumptions are made.

We cannot exclude that the difference between estimated distances and actual distances might have been influenced by some parameters used in our study. For instance, visual distance perception seems to rely on the familiar size of the to-be-estimated object [62]. We adopted an unusual large box to account for the visual angles subtended by the object at far distances. A large object is therefore required to avoid further uncertainty in the distance judgments, especially at greater distances. The size of our box might have affected distance perception *in general*, but not specifically at some head inclinations or vestibular stimulation conditions.

Previous accounts of visual distance perception identified a gravitational bias. Some views treat these as top-down, cognitive biases, which may therefore be post-perceptual. For example, the perceived effort to climb to overcome gravity may lead to the summit of a slope seeming far away [52]. In our experiment these cognitive factors were minimized: in VR there is no actual effort of movement, nor any fear of falling. Our results suggest that upward slope visual distances are overestimated compared to ground level, and we provide novel, causal evidence of why this might be so. We show that gravitational modulation of visual distance perception depends on *on-line* vestibular signals. Previous accounts emphasised memory of past experiences of efforts to overcome gravity, or potential effects of gravity in falling. That is, those accounts treated vertical biases in distance perception as results of prior learning, or as predictions, rather than as on-line modulations of perception. Since concurrent, event-related artificial vestibular inputs boosted visual distance illusions, these illusions may not simply be products of learned and internally-stored contextual associations. Rather, such illusions may owe more to multisensory perceptual integration than has been previously thought.

## Supporting Information

S1 File Data from the experiment in *.csv format. (CSV)
